# Factors Affecting the Repurchase Intention of Organic Tea among Millennial Consumers: An Empirical Study

**DOI:** 10.3390/bs12020050

**Published:** 2022-02-16

**Authors:** Huawei Tian, Abu Bakkar Siddik, Mohammad Masukujjaman

**Affiliations:** 1 College of Economics and Management, Zhoukou Normal University, Zhoukou 466000, China; 2 School of Economics and Management, Shaanxi University of Science and Technology (SUST), Weiyang District, Xi’an 710021, China; 3 Department of Business Administration, Northern University Bangladesh, Dhaka 1213, Bangladesh; masukujjaman@nub.ac.bd

**Keywords:** millennial, organic tea, promotion, repurchase intention, S-O-R theory

## Abstract

The study aims to identify the factors affecting consumers’ intention to repurchase organic tea in an emerging country such as Bangladesh. The study adopted the Stimulus-Organism-Response (SOR) theory, which uses seven constructs as the predictor of repurchase intention. This is a quantitative and empirical study that adopted cross-sectional survey methods. The convenience sampling method was used to collect data from 340 young respondents who visited supermarkets in Dhaka between October and November 2021. In order to analyze the obtained primary data, the structural equation modeling (SEM) approach was used. The findings revealed that product satisfaction, perceived values and brand trust are the predictors of repurchase intention. Surprisingly, we did not find that promotional efforts effected repurchase intention. The study also identified food quality and information quality as the antecedents of perceived value and product satisfaction, while the antecedents of brand trust were product satisfaction, food quality, brand image, information quality and promotional effort. The study suggested numerous theoretical and policy implications to improve repurchase intention of organic tea in the context of emerging economies such as Bangladesh.

## 1. Introduction

Fresh food is a daily necessity, as it provides balanced nutrition for human health. However, the consumption of healthy fresh food is threatened by physical, chemical and biological pollution, resulting in the circulation of pesticide residue-containing and pathogen-harboring fresh produce [1]. As a result, food safety has been a major concern among consumers, food producers and policymakers globally [2]. Food safety has become a critical consumer standard [3] and begins with the elimination of pollutants during the food manufacturing process [4]. Consequently, manufacturers commonly face criticism when the issue of food safety risks is advanced by consumers [5]. In recent years, organic items, particularly organic food, have gained popularity among customers due to their health benefits [6]. Organic foods are safe, since they are grown without herbicides, chemical pesticides, ionizing radiation, bioengineering, sewage sludge, or synthetic fertilizers. Although organic foods are healthier than conventional foods [7], they are inherently more expensive; this constitutes a major deterrent towards their consumption [8,9]. The quality of information labeled on the product has been identified as an important factor in repeat buying, as indicated by studies on mobile banking [10], health infomediaries [11], virtual travel community [12], and food apps sensitization [13]. However, this has not been examined in the context of organic food repurchase intention. Moreover, it is unknown how a customer’s perception of food quality affects product satisfaction, trust and perceived value towards organic food repurchase. As such, there is a need to address this critical knowledge gap by analyzing the antecedent role of product satisfaction, brand trust and perceived value on a customer’s repurchase intentions. 

Repurchase is a result of consumers’ desire to repeat-buy a certain commodity (e.g., organic food) to derive further benefits. However, this is determined by accessibility, household spending capacity and marketing strategies [14]. While existing research has uncovered important indicators of a customer’s behavioral intentions toward organic foods [15,16,17,18], relatively few researchers have examined the determinants of organic food repurchase intentions [19,20,21,22]. Hence, there is undoubtedly a need for an academic analysis of food-related elements that contribute to a customer’s repurchase intentions of organic food products from both theoretical and managerial standpoints. Additionally, understanding the predictive factors that influence a consumer’s repurchase intentions may assist investors and marketers in developing effective strategies in the highly competitive food industry. Studies have shown that repurchase intention differs between generations, especially Gen Y and Gen X [23,24]. Understanding millennials’ green customer behavior is crucial in developing green marketing strategies that target this generation [25,26]. This will also contribute to the comprehensive understanding of millennials’ consumption behavior and the linked issue of sustainable consumption. In Bangladesh, a study has been undertaken on millennial customers’ loyalty in banking [27] and their intention to make green purchasing decisions [28]. Recently, Zheng et al. [29] conducted a study on millennials’ intentions to purchase organic products. However, to the researcher’s knowledge, there is no comprehensive study on millennials’ repurchase intention of organic food in the Bangladeshi context. 

Many studies have examined the S-O-R model in various contexts, such as the effect of price on organic food preference [30], the role of a retail environment on impulse buying [31], online consumer behavior in tourism and hospitality contexts [32,33], consumer behavior in a smartphone context [34], and more recently, organic food consumption intention in the context of Indonesia [35], Japan [36] and Australia [37]. So far, no studies have been conducted on repurchase intention of organic food using the model. Therefore, it is essential to know whether the model is also applicable to the repurchase phenomenon in developing countries such as Bangladesh. By addressing all the above gaps, the current paper seeks to discover the factors that influence the repurchase intention of organic tea products among Bangladeshi Millennials within the S-O-R framework, thereby making significant contributions in terms of novel context, unique outcome and model development.

As a guide, the following sections are organized in chronological order: There is a literature review and the formulation of hypotheses in the second half of the paper. Data collection, sample size, and the survey instrument are all covered in the Section 3. The Section 7 sums up the findings and summarizes the analysis. Ending comments and research suggestions are included in the article’s conclusion.

## 2. Literature Review and Hypothesis Development

### 2.1. The Millennial

Several names have been given to the young generation, namely millennial, Gen Y, Generation Me, or Echo Boomers [38,39]. The name “Baby boomer children” or “Echo Boomers” can be traced to the dramatic rise in birth rates that occurred between the early 1980s and the middle of the 1990s (ages now between 26–41 years). In today’s world, millennials are highly educated, digitally proficient and resistant to traditional marketing tactics. They are also more varied in terms of color and ethnic backgrounds, as well as media consumption, and tend to be more fragmented. Last but not least, these populations are less loyal to brands, as they can access the internet quickly and easily learn new habits, styles and communication methods [40]. A recent study shows that the majority of millennial buyers are excited about buying green items [41,42].

### 2.2. The Stimulus–Organism–Response (S–O–R) Framework

**Figure 1:**
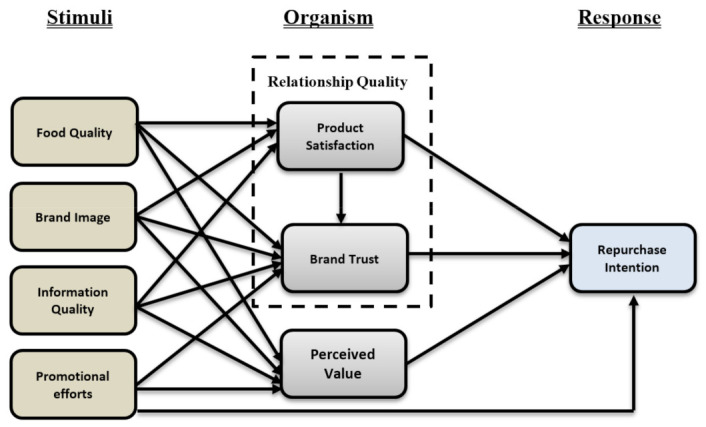
Conceptual Framework.

The S–O–R framework focuses on the interaction between the stimulus, the organism and the response. For example, it can explain certain behavioral patterns, such as why some people become nervous when asked to speak in front of a large crowd, while others are genuinely enthusiastic about the same. The theoretical underpinnings of this work are Mehrabian and Russell’s [43] application of the S–O–R paradigm. In fact, the concept originated in environmental psychology [44] and was derived from behaviorism’s Stimulus-Response (S–R) theory [45]. Animals’ simultaneous reactions to stimuli and responses were found to be the basis for the discovery of the original behaviorism model. If one examines reactions from an S–R perspective, this type of behavior might occur spontaneously as a result of being exposed to particular inputs, such as thinking and emotion [46]. In line with this theory, customers may behave differently depending on their primary emotional reaction to the cues presented to them. This model predicts how customers will react to marketing cues, according to Chen and Yao [47]. For this reason, researchers and marketing managers are collaborating to better understand customers’ reactions and preferences. When it comes to customer satisfaction or dissatisfaction, perceptions of quality (both product and service), perceived value and brand experience are important (as an organism). In either case, the contentment or discontent is what prompts the final reply. Customers’ favorable or negative responses to stimuli reveal their final reaction, i.e., whether they will choose or avoid the brand. Therefore, Figure 1 shows the conceptual framework of the study. 

### 2.3. Hypothesis Development 

#### 2.3.1. Food Quality 

Food presentation, taste, menu variety, healthiness and freshness are all components of food quality [48]. Food quality is a major marketing factor that determines customers’ satisfaction and retention, in addition to providing a positive shopping experience. Food quality can impact customer satisfaction and behavior [49]. Several studies have shown that food quality affects consumers’ happiness [50,51,52,53,54,55]. Additionally, earlier empirical research has suggested that perceived dietary healthiness is critical for customer satisfaction and perceived value [56]. Food safety and quality is also reported to increase brand trust [57] and perceived value [58]. De Toni et al. [22] also noted that perceived food quality influences perceived value of organic product. On this basis, we propose the following:

**Hypotheses** **(H1)–(H3):** *Food quality will positively influence customer satisfaction, brand trust and perceived value*.

#### 2.3.2. Brand Image

Brand image is the sum of personal associations, user experiences and brand beliefs [59]. Eventually, clients will trust a brand more if they have confidence in it. Previous research has shown a positive correlation between brand image and trust [60]. Similarly, brand image has shown to be a strong predictor of consumer satisfaction [61,62,63], whereas others [64,65] found no association. Many studies also showed that brand image affects perceived value [66,67,68]. According to Chen et al. [66], brand linkage increases perceived value, while Nguyen [63] found no relationship for the same. Lai et al. [69] also noted that brand image predicts perceived value and consumer satisfaction. Based on the above argument and empirical evidence, this study proposes the following:

**Hypotheses** **(H4)–(H6):** *Brand image will positively influence customer satisfaction, brand trust and perceived value.*

#### 2.3.3. Information Quality

Information quality measures the usefulness, accuracy and timeliness of information. Consumers may distrust suppliers’ ability and integrity if provided with irrelevant, erroneous, or outdated information. Additionally, they may believe that suppliers will deceive them and disregard their demands. According to Zhou [70], information quality affects users’ trust in mobile websites. Users’ trust in mobile banking, health infomediaries and virtual travel communities have all been found to be influenced by the quality of available information [12].

Customer satisfaction may also be impacted by the quality of information. Quality information saves consumers’ time, helps them compare costs and provides them with relevant sales information [71]. Customers demand reliable, relevant and fast information when purchasing their organic products. Previous research has demonstrated the effect of information quality on users’ satisfaction with mobile internet sites [72], mobile banking [10] and virtual communities [12]. Therefore, it is expected that information quality will also predict consumer satisfaction, brand trust and perceived value for organic products, as dictated in the following hypotheses:

**Hypotheses** **(H7)–(H9):** *Information quality will positively influence customer satisfaction, brand trust and perceived value.*

#### 2.3.4. Promotional Efforts

Promotion (PR) is a type of communication method used by businesses to raise awareness of their products to consumers and differentiate them from opponents [73,74]. A study [75] found a significant influence of promotion on repurchase intention. Promotion encourages non-frequent customers to return and purchase again. Similarly, marketing communication directly influences brand trust [76,77,78]. Since communication builds brand trust, brand communities and customer interactions on social media have been shown to influence brand trust [79]. Promotion enhances consumers’ ties with brand community aspects and boosts brand trust. Additionally, Lee and Ahn [80] discovered that promotions influence customer repurchase intent.

Promotions can serve as a source of information for the evaluation of products and stores ([81]. Unexpected promotions can be attributed to sheer chance and might help alleviate feelings of guilt linked with goods purchased. The researchers discovered that price promotions (e.g., discounts) have a beneficial effect on customers’ estimate of the fair price for the promoted product, their perceived value of the offer, contentment with the purchase and repurchasing intention [82,83].

**Hypotheses** **(H10)–(H12):** *Promotional effort will positively affect brand trust, perceived value and repurchasing intention.*

#### 2.3.5. Product Satisfaction

Satisfaction is a state of mind that develops over time as a result of prolonged positive association with a seller [84]. Customer satisfaction can be described as an overall evaluation of a product or service based on one’s purchase and consumption experience over time [85]. If consumers are dissatisfied with a vendor or its organic products, they may discontinue their patronage. Prior research indicated that contentment is a significant predictor of persistence behavior [72,86,87]. Hence, we anticipated a beneficial influence of satisfaction on consumers’ intent to repurchase organic products.

Numerous empirical studies have demonstrated a positive correlation between satisfaction and trust (e.g., [60,88]. According to these studies, customers’ perception of organic food may be influenced by prior experiences with these items. As a result, it is projected that the ability of organic food to match consumers’ expectation may influence their trust in the food product. In line with this logic, extremely satisfied consumers are more likely to trust organic food. Hence, the following hypothesis 13–14 is proposed:

**Hypotheses** **(H13)–(H14):** *Product satisfaction will positively affect brand trust and repurchase intention.*

#### 2.3.6. Brand Trust

Trust represents an acceptance of vulnerability in anticipation of favorable future behavior of another party. If the concept of customer experience is included, trust is perhaps more crucial for repurchase intention [89], as many experts believe that repurchase intention is dependent on customers’ experiences, which include both cognitive and emotional components, with a particular service provider in the future. According to scholars [90,91], brand trust is the most crucial antecedents of repurchase intention. Hung et al. [87] established a favorable correlation between trusts and repeat buying. Saleem et al. [92] also discovered a favorable association between trust and propensity to repurchase. Thus, we assume that trust will affect the intention to buy organic foods and proposed the following hypothesis:

**Hypotheses** **(H15):** *Trust will positively affect the repurchase intention.*

#### 2.3.7. Perceived Value

Perceived value refers to a detailed assessment of service by customers on the basis of their experience, Zeithaml [93]. Perceived value might influence a person’s desire to revisit a location or repurchase a product. Earlier research has established that perceived value has an effect on a customer’s buying intention [94,95,96] and that the pricing and service quality of air carriers have a substantial impact on a customer’s purchasing choice and perceived value [97,98]. Chen, Li, and Liu [66] discovered that passengers’ perceived value significantly influenced their repurchasing intention, as echoed by Yang et al. [99]. Since organic consumers expect higher product quality and spend more on organic products, their perceived value is expected to influence their repurchase intention, as highlighted in following hypothesis.

**Hypotheses** **(16):** *Perceived value significantly affects repurchase intention.*

## 3. Materials and Methods

A cross-sectional survey method was used in this empirical study. In this regard, the data were gathered solely to ascertain the characteristics of the population at a particular point in time.

### 3.1. Population and Sampling Technique

The participants in the study were individual household members living in Dhaka, the capital city of Bangladesh, and had purchased and drank organic tea items up to the study period. The respondents were gathered from 20 shopping malls and mega shops in 10 locations of Dhaka city and questioned in Bengali, which was later translated into English. The survey was conducted during the period between October and November 2021. The sample size was calculated using G*power for priory sample size sufficiency [100] and to calculate the number of participants required for the study. Cohen [101] recommended a sample size of 153 for seven independent constructs or predictors (effect size f^2^ = 0.15, error type 1 = 0.05, and error type 2 ß = 0.20). Barclay et al. [98] proposed a tenfold sample rule in which they multiplied the maximum number of indicators used in the SEM method by 10. The survey requires 240 (10 × 24) respondents based on these criteria. To alleviate the possible issues associated with small sample sizes, 400 respondents were reached (refusal rate of around 40%) using a non-probability convenience selection strategy. However, 340 samples were selected, while obliterating 37 incomplete and 23 screened-out responses.

Convenience sampling was employed in selecting the respondents [102] for marketing customer data, and the mall-intercept technique was used [103,104] to collect individualized, accurate, and high-quality information [105]. The visitor to the showrooms or centers was requested to participate in the survey. The respondent’s refusal prompted a response from the next most likely candidate. Potential responders were given a questionnaire to fill out and were promised the confidentiality of the information they supplied. For a week in the afternoons, different centers were visited to collect data.

### 3.2. Research Instrument 

The survey instrument was constructed using items from previously validated scales. In this regard, food quality was measured by a three-item scale adapted from Ezgi Erkmen, Murat Hancer, [106]; Sumi and Kabir [107]. Three items for behavioral intention were adapted from Ezgi Erkmen, Murat Hancer, [106]. Information quality was assessed with three items from Gao et al. [108] and Sumi and Kabir [107]. Promotional efforts and brand trust were assessed with three items adapted from Kwon et al. [109]. Product satisfaction comprises three items that were culled from Gao et al. [108], while perceived value and repurchase intention were adapted from Ali and Bhasin, [110]. The responses to the relevant constructs were rated on five-point Likert scales, with 1 indicating strong disagreement and 5 denoting strong agreement. A different set of twenty organic tea customers served as the pre-test sample. In response to the feedback from the pilot sample, minor wording changes were made to the questionnaire to ensure that the scale items were understandable. Table 2 illustrates the items, their convergent validity and their reliability values.

### 3.3. Statistical Analysis

The conceptual model was examined by means of SPSS and AMOS version 21. According to Anderson and Gerbing [111], a two-stage SEM approach was used to analyze the data at hand. The Confirmatory Factor Analysis (CFA) was used in the first stage to evaluate the reliability and validity of the measurement model. The second stage comprised the development of the measurement model. The standardized regression coefficients (β) and *p*-values were calculated for the entire structural model in the second stage in order to examine the overall model fit, in addition to the supposed relationships.

## 4. Results and Discussion

### 4.1. Respondents Profile

According to the survey’s socio-demographic profile, 55% of the 340 respondents were female, 59% were married and 52% were university graduates. Regarding age, all respondents were between 18 and 45 years, indicating that they were millennial customers. As a result, Table 1 displays the socio-demographic data of the respondents.

### 4.2. Reliability Analysis 

The consistency and reliability of the study variables were examined using Cronbach’s Alpha (CA) coefficients and Composite Reliability (CR), as indicated in Table 2. Cronbach’s alpha is a measure that identifies how closely connected a group of items are to one another on a statistical basis. Likewise, composite reliability measures the degree to which the group of constructs contained in the model is related to a specific latent variable. The empirical results revealed that the CA values for all variables vary from 0.769 to 0.903, which exceeds the minimum standard value of 0.7 [112]. Furthermore, the CR of the study variables ranges from 0.760 to 0.850, which is higher than the acceptable limit of 0.7 [112]. Hence, it can be argued that the internal consistency and reliability of the study variables are adequate and satisfactory [113]. 

### 4.3. Convergent Validity

The degree of confidence we have that a trait is well assessed by its indicators is referred to as convergent validity [114]. A high factor loading of measured items indicates that the construct is convergent on a common point, and the values should be greater than 0.7 to be considered acceptable [112]. Consequently, the outputs showed that all factor loadings of measurement items ranged from 0.699 to 0.840 (see Table 1), exceeding the minimum acceptable threshold value of 0.5 [112]. Additionally, the Average Variance Extracted (AVE) and CR can be used to determine the measurement model’s convergent validity, according to Fornell–Larcker [113]. The average variance extracted (AVE) is a measure of the amount of variance that is captured by a construct in relation to the amount of variance due to measurement error. Accordingly, the AVE values range from 0.514 to 0.660, and the CR values from 0.760 to 0.850, both of which are greater than the standard values of 0.5 and 0.7 (Hair et al., 2010; Fornell–Larcker, 1981). Therefore, the scale demonstrated convergent validity.

### 4.4. Discriminant Validity

The Fornell–Larcker criterion and the Heterotrait–Monotrait ratio (HTMT) method were used to evaluate the discriminant validity. The square root of each construct’s AVE score was required to be higher than its highest correlation with other constructs in the model [112] to determine the discriminant validity of the Fornell–Larcker technique [113]. The value of the AVE’s square root in the diagonal surpassed the value of other variables off-diagonal (see Table 2), indicating the existence of discriminant validity [113]. In addition, for robustness, this study examined the HTMT value due to its advantages over Fornell–Larcker in a variety of conditions [115]. The HTMT values were found to be less than 0.90 (see Table 3), confirming that there was no discriminant validity concern [115]. Overall, the results imply that the discriminant validity is adequate and satisfactory.

### 4.5. Testing Normality, Multicollinearity and Coefficient of Determination

Table 4 illustrates the multicollinearity results as well as the study variables’ coefficients of determination. The empirical outputs showed that the skewness and kurtosis values were less than ±3 and ±10, respectively, as shown in Table 3. The results can be considered satisfactory in terms of normality, since the variance derived from the normality testing exhibited no issues [116]. In addition, the VIF approach was used in this study to identify the existence of multicollinearity among the independent variables, as proposed by Kleinbaum et al. [117]. Consequently, the VIF values ranged from 1.794 to 3.230, which fall below the minimum acceptable limit of 10. Therefore, multicollinearity is not a problem in this study. Furthermore, the R–square assesses the model’s explanatory power by finding endogenous factors that are emphasized as determining coefficients. The *R*^2^ value of the endogenous latent variable is considered significant when it exceeds 0.26, moderate when it is 0.13 and weak when it is less than 0.13, as indicated by Cohen [118]. According to the empirical data, the value of *R*^2^ of the endogenous latent construct satisfies under the conditions stipulated by Falk and Miller [119]. As a result, based on the outputs, it can be concluded that the suggested model has a high explanatory power range and is of acceptable quality.

### 4.6. Measurement Model and Common Method Bias

The Confirmatory Factor Analysis (CFA) results were used to verify the measurement model, as outlined by Anderson and Gerbing [111]. In this study, various model fit indicators were used to evaluate the measurement and structural model, as indicated in Table 4. As per the findings, the model fit indices such as χ2/df = 2.285, IFI = 0.938, NFI = 0.919, CFI = 0.943, GFI = 0.913, AGFI = 0.907, TLI = 0.922, SRMR = 0.024, and RMSEA = 0.066 are all within accepted limits [120]. Overall, the model fit is sufficient and acceptable. Furthermore, the single-factor analysis methodology was used to check for common method bias according to Harman’s [121] criteria. Based on the exploratory factor analysis outputs, the single factor explained 47.169% of the variance in the factors, which was less than the 50% threshold, indicating that the common method bias was not existent.

### 4.7. Structural Modeling and Outcomes of Research Hypotheses 

**Figure 2:**
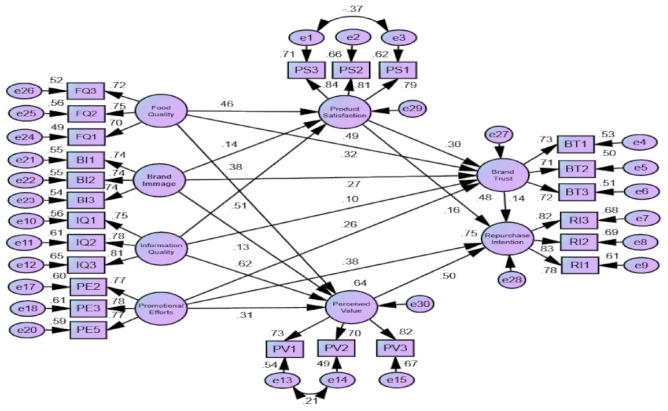
Structural model.

Figure 2 shows the structural model of the study along with standardized estimates. The SEM approach was used to evaluate and confirm the proposed research hypotheses, utilizing multiple models fit indices as depicted in Table 5. As per the empirical outputs, the overall model fit indices were found to be within acceptable limits [120], indicating that the SEM model fit is excellent and satisfactory.

Furthermore, the findings of the SEM and research hypotheses are shown in Table 6. The findings revealed that food quality has a favorable impact on product satisfaction (β = 0.508, *p* = 0.000), brand trust (β = 0.463, *p* = 0.000), and perceived value (β = 0.140, *p* = 0.015), indicating that the H_1_, H_2_, and H_3_ are supported. According to the results, the brand image has a positive effect on product satisfaction (β = 0.619, *p* = 0.000), brand trust (β = 0.313, *p* = 0.000), and perceived value (β = 0.320, *p* = 0.000). As hypothesized, the result showed that the information quality significantly influences product satisfaction (β = 0.298, *p* = 0.003), brand trust (β = 0.382, *p* = 0.000), and perceived value (β = 0.273, *p* = 0.000), thus validating the H_7_, H_8_, and H_9_. Moreover, the promotional efforts exhibit a significant influence on the brand trust (β = 0.129, *p* = 0.023) and perceived value (β = 0.256, *p* = 0.000), suggesting that the H_10_ and H_11_ are supported. Surprisingly, the results showed that hypothesis 12 is not supported, indicating that perceived value has no impact on consumers’ intention to repurchase organic tea products. The results indicated that product satisfaction has a positive effect on brand trust (β = 0.164, *p* = 0.014), thus validating hypothesis 13. Moreover, the association between product satisfaction (β = 0.498, *p* = 0.000), brand trust (β = 0.376, *p* = 0.000), and perceived value (β = 0.136, *p* = 0.041), and a consumer’s repurchase intention towards organic tea has also been found to be statistically significant. As a result, the Hypotheses H14, H15 and H16 are validated. 

Other than the statistical significance, economic significance based on the size of the beta (magnitude of the relationship) is also essential to determine the dominant or passive factors. Product satisfaction (β = 0.498) was the most influential factor influencing repurchase intention for organic tea, followed by brand trust (β = 0.376). The most passive factor was the promotional effort (β = 0.102) on the repurchase intention of organic tea. The highest relationship was found between brand image (β = 0.619) and product satisfaction, while the lowest was information quality (β = 0.298). The strongest predictor of perceived value was brand image (β = 0.320) and the weakest was food quality (β = 0.140). Likewise, food quality was the highest predictor (β = 0.463) of brand trust and promotional effort was the lowest (β = 0.129).

## 5. Discussions and Conclusions

Based on the SOR paradigm, the study successfully revealed the factors impacting the repurchase intention of organic tea products in the Bangladeshi context. The links between stimuli and organism were used to examine the effect of brand name on store image and the effect of promotion on perceived value. The study used the organism-response model to explain the relationship between perceived value, brand image, and behavioral intentions. According to the outcomes of the study, food quality has a favorable impact on product satisfaction, brand trust, and perceived value, indicating that the H_1_, H_2_, and H_3_ are supported. This result is in agreement with past studies [22,56] and implies that the higher the food quality perceived by the consumers, the higher their satisfaction, trust and perceived value for repurchasing organic tea. On the other hand, the findings of the study indicated that the brand image has a positive effect on product satisfaction, brand trust and perceived value. Hence, Hypotheses H4 to H6 are accepted, confirming the earlier studies [60,63] in a similar context. This suggests that brand image of organic tea promotes brand trust, satisfaction and perceived value, thereby resulting in repurchase intention of the consumers. 

Based on the empirical results, the information quality significantly influences product satisfaction, brand trust, and perceived value, thus validating the H_7_, H_8_, and H_9_. These findings corroborate the outcomes of past studies [12,70,72] and suggest that the greater the quality of information presented on the organic tea label, the greater the chances of acquiring satisfaction, trust and value, which eventually promote the repurchase intention of organic tea. As expected, the promotional efforts exhibit a significant influence on the brand trust and perceived value, suggesting that the H_10_ and H_11_ are supported. As a result, this outcome further advocates the findings of Orzan et al. [76] and Lee and Ahn [80] from the perspective of e-commerce. A promotional campaign reminds customers about the product, which sparks positivity in terms of product satisfaction and trust and consequently leads to repurchase intent. 

Surprisingly, the results showed that hypothesis 12 is not supported, indicating that perceived value has no impact on consumers’ intention to repurchase organic tea products. This is in contrast to the studies [66,127] and consistent to findings of the study by Correa [128], which observed a positive relationship between perceived value and repurchase intention. This outcome could be explained in two ways: promotions can be used to bring in new customers and entice them to make their first purchase, as well as to persuade existing customers to move to your brand from another [129]. While promotions encourage infrequent consumers to return and increase their likelihood of making another purchase, brand loyalty customers are more likely to make another buy, regardless of the promotion [130]. Secondly, there might have been a mediating role of brand trust in the relationship between promotional efforts and repurchase intention. 

Confirming the past studies [60,88], the results indicated that product satisfaction has a positive effect on brand trust, thus validating hypothesis 13. This means that a high level of satisfaction raises customers’ levels of trust, thereby resulting in a better relationship of customers with the organic product vendors. Moreover, the association between product satisfaction, brand trust, and perceived value, and a consumer’s repurchase intention towards organic tea has also been found to be statistically significant. As a result, the Hypotheses H14, H15 and H16 are validated, which is in agreement with the past scholarly studies [66,86,92]. Therefore, it can be concluded that product satisfaction, perceived values and brand trust predict repurchase intention. Interestingly, we did not find promotional efforts to effect repurchase intention. The study also found that the antecedents of perceived value and product satisfaction are food quality and information quality, while the antecedents for the brand trust were product satisfaction, food quality, brand image, information quality and promotional effort.

## 6. Implications of the Study

### 6.1. Theoretical Implications

The study offers a number of theoretical contributions to the literature. First, the study complements the present theory with new results. Information quality is commonly used as a construct in various technology adoption studies and has been integrated with the repurchase intention model in this study. The result established that information quality is an important predictor of customer satisfaction, trust and perceived value in the organic tea product context. Second, the study also revealed that promotional efforts are not directly related to repurchase intention; rather, it is valuable in combination with brand trust, satisfaction and perceived values, thus challenging existing empirical thoughts. Hence, this study extends the body of knowledge in the organic tea repurchase intention by validating existing links and adding new outcomes. Third, the study provides a good fit model with greater explanatory power of about 75% for repurchase intention, which is almost similar to the contemporary studies [19,21,22] on organic tea repurchase. This will certainly update the present literature applying this model and scales. Fourth, according to the S-O-R theory, an external stimulus (food, information, or promotional efforts) affects an organism (product satisfaction, brand trust, or perceived value), which in turn determines reaction (repurchase intention). The S-O-R framework on organic food purchase and repurchase intention in the Bangladeshi perspective is absent. Therefore, our study adds to the body of knowledge on organic food consumption behavior by looking at how different hypotheses complement one another in explaining why customers return to buying organic food again and again. Fifth, the respondents in this study constitute primarily the younger generation. According to Owens and Nowell [131], young consumers demonstrate a preference for emotional appeal over rationality, highlighting the necessity for this age group to be examined independently in order to identify emotional appeals, particularly in relation to consumer behavior in Bangladesh. Ultimately, the study provides a clearer picture of the attitudes of today’s young consumers toward organic tea consumption in the future, and also adds to the growing body of knowledge about Generation Y as both consumers and citizens. Finally, based on the authors’ knowledge, this is the first study to examine repurchase intentions from an organic food context and the second in the domain of organic tea products following Sumi and Kabir’s [107] study.

### 6.2. Practical Implications

The results of this study have important managerial implications for companies to attract customers. First, this study guides managers and policymakers in the organization on the reasons for infrequent buying of organic tea products. For example, the study found that product quality is an important determinant of consumer satisfaction, trust, perceived value, and ultimately, repurchase intention. Quality assurance is a separate department in most organization and can play a pivotal role in ensuring product quality as specified. When there is a synergy between quality and price of a product, customers tend to purchase the product repeatedly. Thus, managers and policymakers must ensure proper quality of products to lower promotional expenditure and other efforts. Companies can also promote the quality of the product (organic tea) to customers by demonstrating the processes involved in making such products in the mall or in mass media. 

Second, the study found that promotional efforts do not directly impact the repurchase of organic tea products, but do indirectly via trust, satisfaction and perceived value. This does not mean that companies should not advertise or promote their products. It only indicates that sales promotion or promotional campaign does not ensure repurchase. From the study’s findings, product satisfaction was found to have a greater effect on repurchasing compared to other predictors, thus accentuating the necessity of maintaining the highest quality and assurance of support. Moreover, managers must rethink the trustworthiness of promotional efforts, the product quality, and information quality to ensure better advertisement content. As information quality is found related to customer satisfaction, trust, and perceived value, managers and policymakers must be careful about the information attached to the label in terms of accuracy and adequacy. Policymakers in this sector should also control the unregulated use of organic labeling for promotion purposes only. Efforts from certified organizations could also restore trust of organic tea products. 

Third, it is noteworthy that perceived value exhibits a positive influence on repurchase intention. Furthermore, perceived value is intricately linked to food quality, brand image, and promotional efforts, all of which may result in adverse influence on repurchase intention if ignored. Hence, it is vital for organizations to cut down the price of organic tea products, probably by scaling up their productions. The government should also provide financial and policy support towards organic food production to strengthen the industry and ensure public access to the product at a cheaper price. 

## 7. Study Limitations and Future Research 

The major limitation in this study is the use of self-report instruments, which could result in a common method bias in measuring the variables [132,133]. However, the probability of common biases was reduced in this study by separating the instruments and motivating the participants. Additionally, data were obtained in Bangladesh. To generalize the findings, research across countries and cultures is required. Finally, future studies may also consider integrating different factors (e.g., word of mouth, brand loyalty) into the model. The study did not integrate any mediating relationship, which could be tested in future research with constructs such as trust with behavioral intention, product satisfaction, food quality, information quality, and repurchase intention. The study only considered the buying intentions of young consumers, missing the outcomes of other generations such as X and Z. The study could be extended by conducting a separate study for those generations or could be on a comparative basis between generations. However, the same study can be replicated by incorporating the repurchase behavior of organic tea into the model. In particular, future research could incorporate the transition of repurchase intention into repurchase behavior with a longitudinal research design.

## Figures and Tables

**Table 1:** Respondents’ profile.

Aspects	Classification	Frequency	Percentage (%)	Aspects	Classification	Frequency	Percentage (%)
Gender	Male	187	55	Education	No formal education	0	0
	Female	153	45		Higher Secondary or below	61	18
Age	15–20	5	1.5		Graduate	177	52
	20–25	61	18		Postgraduate and above	102	30
	25–35	139	41	Marital Status	Married	201	59
	35–40	97	28.5		Single	139	41
	40–45	38	11				

Source: Authors’ calculation.

**Table 2:** Reliability and validity analysis.

Constructs	Standardized Loading	Conbach’s Alpha	CR	AVE
Food Quality (FQ) [106,107]		0.769	0.766	0.522
FQ1: Organic tea products are tastier	0.699			
FQ2: Organic tea products have no hazardous (natural) materials	0.746			
FQ3: Organic tea products offer a variety of options for good flavors	0.722			
Brand Image (BI) [106]		0.780	0.784	0.548
BI1: The brand of organic tea products has better characteristics than that of the traditional tea products	0.745			
BI2: The brand of organic tea products has a reputation for quality	0.738			
BI3: The brand of organic tea products is familiar to me	0.738			
Information Quality [107,108]		0.826	0.822	0.607
IQ1: The product provides me with sufficient information about my needs.	0.748			
IQ2: I receive accurate information from the product lebel.	0.782			
IQ3: Labeling on organic tea is clearly understandable	0.806			
Promotional Efforts [109]		0.819	0.819	0.601
PE1: The marketing of the brand of organic tea that I choose leaves me with a positive impression	0.774			
PE2: Promoting my favorite organic tea brand makes me happy.	0.782			
PE3: Promoting my organic tea brand brings good memories.	0.770			
Product Satisfaction [108]		0.862	0.854	0.660
PS1: I feel satisfied with the product attributes	0.787			
PS2: I feel satisfied with the product information on the labels	0.810			
PS3: Compared to traditional tea products, I am satisfied buying organic tea.	0.840			
Perceived Value (PV) [110]		0.865	0.795	0.566
PV1: Organic products purchased are a good buy.	0.732			
PV2: Consuming organic food has a high overall value.	0.699			
PV3: High price of organic tea creates a great value to me	0.820			
Brand Trust [109]		0.826	0.760	0.514
BT1: I always trust my favorite brand of organic tea product	0.725			
BT2: My favorite brand of organic tea never disappoints me	0.708			
BT3: Certification of organic tea of my favorite brand is highly reliable	0.717			
Repurchase Intention [110]		0.903	0.854	0.660
RI1: I intend to recommend organic tea products to my neighbors	0.783			
RI2: I will keep buying the organic tea product in the future.	0.832			
RI3: I intend to purchase organic tea products in the future.	0.822			

Note: CR = Composite reliability, AVE = Average variance extracted, Source: Authors’ calculation.

**Table 3:** Correlation of latent variables and square roots of AVE.

Variables	FQ	BI	IQ	PE	PS	PV	BT	RI
Food Quality (FQ)	**0.722**							
Brand Image (BI)	0.595 **	**0.740**						
Information Quality	0.486 **	0.425 **	**0.779**					
Promotional Efforts	0.485 **	0.476 **	0.388 **	**0.775**				
Product Satisfaction	0.566 **	0.471 **	0.566 **	0.563 **	**0.812**			
Perceived Value (PV)	0.557 **	0.504 **	0.664 **	0.568 **	0.735 **	**0.752**		
Brand Trust	0.576 **	0.536 **	0.472 **	0.541 **	0.620 **	0.557 **	**0.717**	
Repurchase Intention	0.646 **	0.499 **	0.492 **	0.682 **	0.711 **	0.750 **	0.628 **	**0.812**
Mean	3.141	3.391	3.669	3.750	3.341	3.452	3.295	3.414
Standard Deviation	0.701	0.647	0.654	0.647	0.819	0.783	0.688	0.782
Skewness	−0.288	−0.519	−0.267	−0.406	−0.207	−0.313	0.126	−0.214
Kurtosis	−0.291	0.236	0.114	0.158	0.175	−0.343	−0.131	−0.153

Note: (In Table, bold elements, the square root of AVE). ** indicates that correlation is significant at the 0.01 level (2-tailed).

**Table 4:** Heterotrait–Monotrait Ratio (HTMT).

Variables	FQ	BI	IQ	PE	PS	PV	BT	RI	VIF	*R* ^2^
Food Quality (FQ)									2.189	
Brand Image (BI)	0.794								1.794	
Information Quality (IQ)	0.603	0.530							1.916	
Promotional Efforts (PE)	0.603	0.595	0.473						2.008	
Product Satisfaction (PE)	0.698	0.573	0.677	0.675						0.49
Perceived Value (PV)	0.514	0.612	0.784	0.675	0.829				3.230	0.64
Brand Trust (BT)	0.714	0.666	0.569	0.658	0.742	0.659			1.996	0.48
Repurchase Intention (BI)	0.788	0.608	0.589	0.819	0.810	0.802	0.750		3.062	0.75

**Table 5:** Results of CFA and structural model with standards.

Fit Indices	Values for CFA	Values for Structural Model	Standards with Sources	
χ2/df	2.285	2.539	<3	[122]
IFI	0.938	0.923	>0.900	[120]
NFI	0.919	0.911	>0.900	[120]
CFI	0.943	0.923	>0.900	[123]
GFI	0.913	0.905	>0.900	[120]
AGFI	0.907	0.901	>0.900	[124]
TLI	0.922	0.906	≥0.90	[125]
SRMR	0.024	0.027	<0.080	[120]
RMSEA	0.066	0.073	<0.080	[125,126]

Source: Authors’ calculation.

**Table 6:** Structural model and hypothesis testing result.

Hypotheses	STD Beta	STD Error	*t*-Values	*p*-Values	Significance (*p* < 0.05)
H1: FQ→PS	0.508	0.075	7.921 ***	0.000	Significant
H2: FQ→BT	0.463	0.079	6.893 ***	0.000	Significant
H3: FQ→PV	0.140	0.076	2.433 **	0.015	Significant
H4: BI→PS	0.619	0.068	9.487 ***	0.000	Significant
H5: BI→BT	0.313	0.061	5.365 ***	0.000	Significant
H6: BI→PV	0.320	0.081	3.738 ***	0.000	Significant
H7: IQ→PS	0.298	0.080	3.022 ***	0.003	Significant
H8: IQ→BT	0.382	0.065	6.149 ***	0.000	Significant
H9: IQ→PV	0.273	0.074	3.995 ***	0.000	Significant
H10: PE→BT	0.129	0.067	2.273 **	0.023	Significant
H11: PE→PV	0.256	0.061	4.001 ***	0.000	Significant
H12: PE→RI	0.102	0.077	1.257	0.209	Not Significant
H13: PS→BT	0.164	0.059	2.458 **	0.014	Significant
H14: PS→RI	0.498	0.070	7.019 ***	0.000	Significant
H15: BT→RI	0.376	0.061	6.385 ***	0.000	Significant
H16: PV→RI	0.136	0.072	2.048 **	0.041	Significant

** Significant at 5% level, *** Significant at 1% level.

## Data Availability

The data that support the findings of this study are available from the corresponding authors upon reasonable request.
